# Mycelium-Based Composite: The Future Sustainable Biomaterial

**DOI:** 10.1155/2022/8401528

**Published:** 2022-03-12

**Authors:** Digafe Alemu, Mesfin Tafesse, Ajoy Kanti Mondal

**Affiliations:** ^1^College of Biological and Chemical Engineering, Department of Biotechnology, Addis Ababa Science and Technology University, Addis Ababa-16417, Ethiopia; ^2^Center of Excellence for Biotechnology and Bioprocess, Addis Ababa Science and Technology University, Addis Ababa-1641, Ethiopia; ^3^Institute of Fuel Research and Development, Bangladesh Council of Scientific and Industrial Research, Dhanmondi, Dhaka-1205, Bangladesh

## Abstract

Because of the alarming rate of human population growth, technological improvement should be needed to save the environment from pollution. The practice of business as usual on material production is not creating a circular economy. The circular economy refers to an economic model whose objective is to produce goods and services sustainably, by limiting the consumption and waste of resources (raw materials, water, and energy). Fungal-based composites are the recently implemented technology that fulfills the concept of the circular economy. It is made with the complex of fungi mycelium and organic substrates by using fungal mycelium as natural adhesive materials. The quality of the composite depends on both types of fungi and substrate. To ensure the physicochemical property of the fabricated composite, mycelium morphology, bimolecular content, density, compressive strength, thermal stability, and hydrophobicity were determined. This composite is proven to be used for different applications such as packaging, architectural designs, walls, and insulation. It also has unique features in terms of low cost, low emission, and recyclable.

## 1. Introduction

The world population is booming at an alarming rate. It needs technological improvement to meet the needs of the people unless it leads to environmental pollution, waste generation, and natural resource depletion. With rapid population growth and urbanization, annual waste generation is expected to increase by 70%, from 2.01 billion tons in 2016 to 2.2 billion tons and 3.40 billion tons in 2025 and 2050, respectively [1, 2] (The World Bank, 2019). The major sources of these wastes are from commercial centers, construction sectors, domestics, agriculture, and industries [3]. Improper recycling of wastes generated from these sources has resulted in the pollution of water bodies, air, landfills, and fertile soils [4]. To minimize the environmental effect of such wastes, recycling technology will be the prominent solution. Natural resources are getting scarce, necessitating a search for renewable and recyclable materials, and alternative ways for using existing resources are the other responsibilities of human beings.

As the world human population increases, so does the need for industrialization and natural resource depletion. About 66% of the world population will live in urban areas by 2050 according to the United Nations prediction [5]. Similarly, the need for housing, infrastructure, packaging, and other industrial products will be increased. The practice of such material production is one of the causes of environmental pollution [6]. According to IPCC data presented in 2010, about 18% of the global greenhouse gas (GHG) emissions could be from production, transportation, and demolition of materials [6].

Most industrially fabricated materials such as construction and packaging materials nowadays are nonrecyclable and environmentally unfriendly. The utilization of these conventional materials consumes energy, limits natural resources, and pollutes air, soil, and water bodies during production, transportation, and demolition. Eight to ten percent of the global total carbon dioxide emissions were released from the manufacturing of construction materials [7].

The practice of business as usual in material production will not create a sustainable environment and circular economy. The advancement of technology in sustainable materials production has become one of the most important key issues in the field of biotechnology and civil engineering research. Recent studies point out that there is a possibility of biocomposites production from the mycelium substrate complex to replace the conventional materials [8, 9]. This biomaterial or composite is grown rather than manufactured [9]. Mycelium is the vegetative part of a fungus with the mass of branching hyphae and a hollow and tubular structure that provides a fast-growing, safe, and inert material as the binding matrix [10]. It acts as a natural binder, where it fastens onto any organic substrates around it (i.e., coffee husk, sawdust, wheat bran, straw, and bagasse) to create a superdense network of threads [11, 12]. Mycelium-derived materials have several key advantages over conventional synthetic materials including their low cost, low density, ecofriendly nature, and energy consumption [13, 14]. The main aim of this review is to elaborate on the possibility of fungal mycelium for the production of various sustainable materials and to verify the future prospective.

## 2. Literature Review

### 2.1. Sustainable Materials

Materials that produce less pollution and waste during manufacturing, utilization, transportation, and demolition process as well as economically feasible are being considered as sustainable materials [15]. Most conventional construction materials are nonrecyclable, consume high energy, are environmentally unfriendly, and require high cost. When such material enters the environment, it will remain there for many years [11]. Most conventional industrially fabricated materials are the largest energy consumers and greenhouse gas emitters, both in developed and developing countries [16]. In developing countries, only the production of construction materials accounted for 32% of total global energy consumption and 19% of greenhouse gas (GHG) emission in 2010, and this result will be doubled or tripled in the next 50 years [17]. While in developed countries such as the US, from all industrial-related emissions in 2002, about 6% was from construction sector [18]. Projection stated by the same author also predicted that about 80 million metric tons of CO_2_ will be emitted from the construction sector in 2030 [18].

Most construction materials such as blocks, partition walls, insulation, and concrete in the world are made up of cement, gypsum, sand, metal, and wood products. About 4.18 million tons of cement were produced globally in 2014 for these purposes [19]. If the perception of using cement as construction materials is not changed globally, then 3.5 billion metric tons of cement could be produced in the world by the year 2050 [20]. Among all building materials, cement is the most widely used, plays a crucial role in the construction sector, and attains special features in the construction activities due to its durability, high compressive strength, and resistance to chemical and weathering actions [21, 22]. Not only these different wood products, but also gypsum and polymers are used in the construction sector. Despite their high strength and resistance to weather conditions, these materials have a great role in environmental pollution from production to demolition due to their nonbiodegradable feature and high emission. Using wood products for construction purposes leads to deforestation and unexpected weather fluctuation. According to the findings of [23], the main cause of Ethiopian forest reduction is utilization of woods for construction purposes. To keep the world clean, sustainable activities such as the use of (a) recyclable materials, (b) locally available materials in order to minimize transportation cost and fuel, (c) ecofriendly materials, and (d) cost-effective materials and (e) materials design improvement should be carried out.

The application of microorganisms in biomaterial production especially in the construction and packaging sector is the anticipative technology in the near future to bring environmental sustainability [14]. The concept of using mycelium as a material was started in 2007 [24], by the Evocative company owners Eben Bayer and Gavin McIntyre [25]. This company produces high-quality packaging products that can be 100% recyclable and nontoxic [26]. Microbes can be applied in the construction sector through two major directions: (1) indirect method, by the production of construction materials by using enzymes extracted from microbes, and (2) direct method, direct application of microbes such as cell wall, mycelium, and spore of microorganisms [27]. An enzyme extracted from microorganisms is used for soil stabilization. Some other microbes also precipitate calcite from their cell wall and are used for calcium carbonate production. As illustrated in Table 1, microbes have a great role in the production of construction materials such as bioconcrete, bioblock, biocement, and biopolymer through precipitation of their calcium carbonate, secreting soil stabilizing enzyme, and through their unique natural adhesive property by their mycelium [28–30].

Bio-based materials combine many mitigation strategies such as low embodied energy and carbon, low cost, recyclable, use locally available materials, and are available as waste and byproducts; as a result, they can be easily integrated with the prefabricated constructive system [25]. In addition, bio-based construction materials are better in thermal resistance, ease of production, attractive, and self-growing rather than manufacturing [11, 25, 44–48]. Raw material availability and ease of production for microbe-based materials result in cost minimization. Using biomaterials can reduce costs about 80 times lower than conventional materials [49]. Biological construction materials can reduce carbon emissions nearly by 800 million tons per year [50]. If immediate action cannot be taken to replace conventional materials such as cement, gypsum, and other polymer and plastic products with biomaterials, it will be very difficult to withstand its environmental impact. In the same manner, biological materials have also indirect advantages in organic waste reduction because most raw materials used for the production of microbial-based materials are locally available organic wastes.

The cost was the prior advantage of the mycelium-based block (MBB) over conventional materials. Mycelium-based blocks are 80 times cheaper than cement- and gypsum-based blocks [49]. The author points out that only 18.92 USD is needed per m^3^ of MBB, whereas 936.87 USD per m^3^ was needed for the cement-based block. Apart from these inherent physicochemical properties of these bioblocks, the additional and most significant benefits are the green synthesis approach, ease of fabrication, nontoxicity, and biodegradability.

### 2.2. Mycelium as a Biomaterial Production

**Figure 1:**
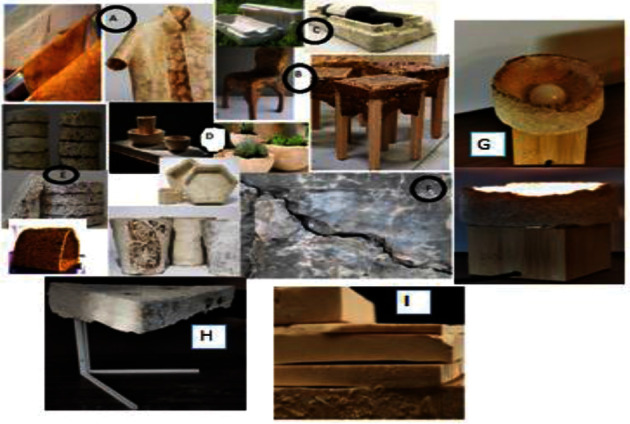
Different mycelium-based materials [8, 13, 24, 30, 33, 58, 62]. Leather-like materials, with companies in Indonesia, Italy, and the United States having already released promotional material and prototypes in fundraising campaigns, and they are twice cheaper than convectional materials (a). Lightweight mycelium-based composites, despite their load-bearing capability and durability, led the designers to explore designing various furniture by cultivating mycelium, such as chairs (b). Mycelium-based packaging as an environment-friendly alternative to plastic-based foam packaging (c). Light and low-density kitchenware and pots as biodegradable and recyclable alternatives to single-use plastics (d). Blocks made of mycelium substrate complex, partition wall, and indoor construction (e). Fungus used for self-healing of cracks through calcite production (f). Mycelium-based light fixture to enhance lamp light reflection (g). Coffee table with mycelium-based tabletop (h). Flexible mycelium-based polymer-like material (foam) (i).

Mycelium has been used for a long period in medicinal industries and molecular compounds [51]. It has been used as the production of dietary supplements or nutraceuticals such as antitumor, antimetastatic, antioxidant, anti-inflammatory, insecticidal, and antimicrobial. Gradually, utilization of mycelium was translated into mycoremediation since the 1980s [52–54]. Beyond bioremediation and medicinal application, nowadays mycelium is applied in biomaterial production such as biocement, bioblock, and bioenzyme. A few companies such as MycoWorks (https://www.mycoworks.com, 2021), NEFFA [55], Evocative Design [56], and MOGU [57] started to design and commercialize mycelium-bound composites in the world. Since 2007, designers and architects started to use mycelium-based products such as synthetic leather [33], kitchen utensils [8], packaging items [25], various furniture [58], wall and ceiling panels [4], biocement [31, 34, 59], and blocks and masonry units [8, 45, 49, 60, 61] as alternatives to conventional materials.  Figure 1 shows different mycelium-based materials. Of all materials, synthetic leather is made up of pure mycelium, while packaging items, furniture, panels, and blocks are made of a combination of mycelium and organic substrates [33, 63]. Using mycelium-based material as an alternative to polystyrene and plastic packaging was started in 2013 by a company called Evocative Design [56]. As observed in the figure, fungal-based composites can replace conventional materials. Different artifacts can also be produced because they can be molded into different shapes and with low density.

Mycelium is a dense network of thin strands called hyphae that grow and fuse together into a solid material. Mycelium growth forms self-assembling bonds and miles of tiny white fibers which invade and degrade the organic substrate, gradually colonize the organic matter, and bind them into strong and 3D structure materials [11]. During mycelium colonization, the cellulose or lignin or both compounds of the substrate can be degraded by fungi through secreting an enzyme such as lactase, lignin peroxidase (Lip), and manganese peroxidase (MnP) [64], whereas hemicellulose is usually attacked by all the species [65]. Not all species have the same lignin-degrading ability. While degrading lingocellulose substrate, the mycelium can assemble together and form a block-like structure. This self-assembling property of mycelium makes fungi unique in the production of noble bioproducts. As mycelium can grow easily on organic wastes, its derivative materials have the potential to become the material of choice for a wide variety of applications because they are emission-free, recyclable, and of low cost [11]. Mycelium-based materials (MBm) are recyclable and renewable and can substitute other conventional materials [66]. These materials are fully biological so that they can be selected by different designers and architects to be used for packaging and building industries with little or no cost and environmental damage [67]. Mechanical and hydrophilic properties are some of the drawbacks of MBm; however, different recent studies show that these properties could be adjusted by improving production methodology, best fungal and substrate selection, and strain gene modification [49, 60].

Species selection is one of the most challenging tasks for different researchers in effective biomaterial production. Criteria for species selection include mycelium density, growth rate, cost of growth media (substrate), noxiousness level [32], ease of cultivation, and mycelium structure [68]. Fungi have dense mycelium, grow fast at locally available media, and have no toxicity level. Phylum Basidiomycota is selected for biomaterial production by different scientists due to their mycelium natural adhesive property and their ability to degrade lignocellulose [33]. This phylum has been selected due to the presence of two important features: septa and anastomosis [67]. (1) Septa are special transverse cell walls of fungi having an opening valve that can be closed, help the cell to decrease the damage caused to the colony by a rupture through on and off the opening, and also greatly increase the robustness of the mycelium [66], whereas (2) anastomosis has a special feature in making two different hyphae to fuse together when they meet [32, 68]. When two or more hyphae are fused together, a large network can be formed and it allows for more nutrient transportation between the substrate through the large network; as a result, mycelium can grow fast, strong, and dense. These two hyphae structures make Basidiomycota unique in biomaterial production.

*Pleurotus ostreatus (P. ostreatus*) and *Ganoderma lucidum (G. lucidum*) are found to be the most common species that belong to the phylum Basidiomycota [32, 44, 49, 60]. These species belong to the same class of Agaricomycetes and have a different order, family, and class. *Pleurotus ostreatus* is categorized under order Agaricales, family Pleurotaceae, and genus *Pleurotus*, while *Ganoderma lucidum* goes to order Polyporales, family Polyporaceae, and genus *Ganoderma* [64]. Fungal species that belong to the order Agaricales (*Pleurotus ostreatus*) result in the production of higher compressive strength biomaterials and have more stiffness properties [33]. That is due to its ability to colonize and grow rapidly on various organic materials containing lignin, cellulose, and hemicelluloses and thick cell walls [4, 44, 61]. *P. ostreatus* is a member of oyster mushroom, sometimes known as “white oyster” (Precious, 2019), and the most widely utilized species worldwide for enhancing food security.

This species can colonize and degrade a large area of lignocellulose waste streams such as sawdust and straw within a few days [32, 33]; furthermore, it has rough skin and a more rigid appearance [63]. As a result, most researchers prefer this species for its best properties of biomaterial production.

The species type, substrate type, and manufacturing methods play a great role in the quality of mycelium-based materials [64, 68, 69]. However, the effect of fungal species on final material properties is more dominant than the effect of substrate type [33]. Biomaterial quality is greatly depending on fungal species type rather than other factors. That is due to the presence of chitin in the fungal mycelium which has a prominent role in substrate adhesion. The following factors should be considered for substrate selection: (1) nutritional content, (2) availability and abundance, (3) degradability, (4) cost, (5) textural and structural properties, and (6) compatibility [68]. Substrate nutrient such as glucose is the main source of nutrient for fungi; to get this nutrient, some fungi break down cellulose into glucose. Substrates with high cellulose content allow fungi to grow rapidly; as a result, it corresponds to a high tensile strength [68]. That is in fact due to higher mycelium density and chitin content. However, some plant species such as hemp secrete a toxic substance which is incompatible with fungal growth [70]. Such plant species should be selected to save the strain life. The most known substrates for the production of mycelium-based materials are wood chips [30, 32, 66], sawdust, straw [4, 8, 9, 33, 71], coconut powder [72], garden waste [14], and bagasse [4]. These substrates are selected due to their compatibility for fungal growth and their lignocellulosic content. However, the mycelium invasion rate and biomaterial quality vary from substrate to substrate.

The mycelium growth rate in the straw substrate is faster than in sawdust [8]; similarly, mycelium growth on bagasse shows a faster growth rate than sawdust and its mixture [4]. This is due to the nutritional variation and complexity of glucan in sawdust. In addition, straw and bagasse have softer particle properties than sawdust, so the fungi can utilize nutrients easily from soft substrates than hard substrate according to [32]. To enhance the nutritional content, different supplements such as wheat bran [8]and rice bran and different agricultural straw [72] are mixed.  Table 2 shows different mycelium-based materials with various strains, supplements, and substrates. Fungi with high mycelium development on the substrates result in relatively higher MBm compressive strength [8]. That is because the substrate mixed with the supplement shows higher mycelium growth than the nonmixed substrate.

## 3. Mechanisms

MBB production consists of the following six major stages: strain cultivation, substrate preparation and sterilization, substrate inoculation, molding, deactivation, and transportation [28, 30, 48, 79]. Strain cultivation is started from culturing, isolation, and screening. Most Basidiomycota strain culture begins from the tissue culture method or spore print method. In addition, they are also isolated from dead trees, soil, and other organic wastes [80]. The optimum temperature and humidity for most fungi mycelium development are 25–30°C [26] and 60–65% [81]. The temperature and humidity below and above the optimum level reduce the mycelium growth rate or damage the strain. The obtained pure culture is inoculated into grains for spawn production [82, 83]. Spawn is the grain inoculated with pure culture used for startup of the substrate colonization. Most of the time, it can be prepared by using grain and sawdust filled into glass bottles or polyethylene plastic bags. Substrate colonization rate is determined by the amount of inoculum [84], types of strain used, and types of substrate [4]. Amount of optimum spawn used for inoculum varies in different studies: 10%–20% [28], 10% [82], 3% [32], and 15% [68] in dry weight basis. As the amount of inoculum increases, the growth rate increases and the contamination level decreases [84]. The high amount of inoculum can occupy a large surface of the substrate so that the mycelium can fully colonize within a short time. As a result, the chance for contamination is less. However, the extended amount of inoculum may affect the biomaterials' quality.

**Figure 2:**
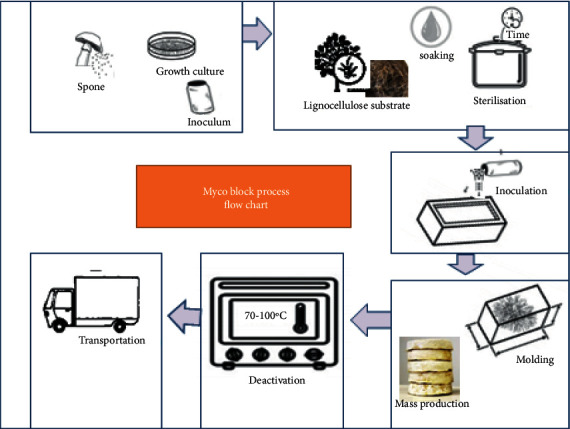
Flowchart showing production of mycelium-based composite (adopted from [28]).

Mycelium development is evaluated by chemical and physical parameters such as visual inspection, pH test, organic matter content, water content [32], and mycelium surface morphology [4]. Well-developed fungal mycelium has decreased pH level and total organic matter which is due to enzymatic digestion, whereas the amount of nitrogen and water increases as mycelium is well developed [30, 32]. As mycelium grows, a network of branching hyphae composed of biomolecules mainly chitin grow out of the substrate into the air creating a fluffy or compact layer (fungal skin) covering the substrate and a vast three-dimensional matrix [10, 69, 76]. The mycelium (vegetative part) can be grown into dense material in a mold to form different shapes for different items. While growing, the mycelium adheres to the substrate and can be shaped to different structures or new design objects [11]. Once reaching the desired structural characteristics, the fungal growth is stopped from further growth [8, 65, 84]. Fungal growth can be stopped by drying and/or heating the colonized substrate. However, drying cannot stop mycelium growth permanently. It makes the strain preserve the fungi in a “hibernated” state. In the latter case, growth can be reinitiated under suitable environmental conditions [33]. Heating the mycelium deactivates the strain from reinitiation and stops its growth permanently. In addition to the deactivation of the cell, heating also helps to detoxify the strain in case the strain is toxic.  Figure 2 shows the major process flowchart for the production of mycelium-based materials.

Table 3 shows the comparative study of mycelium-based block with the conventional one in terms of density, strength, cost, recyclability, and persistency. The mycelium-based composites are shown to be of low dense and low cost, recyclable, and can be made with locally available raw materials.

### 3.1. Factors Affecting Mycelium-Based Materials

Different factors can affect the quality of MBm including strain type [44], substrate type [4], mycelium growth condition, incubation time [74], additive used [8], fabrication method [60,63], and types of inoculum used [65]. Better growth of mycelium on the substrates provides a higher compressive strength of MBm. An increase in the incubation period and pressing time also affects the strength of the materials. Most authors agreed that heat press can increase the tensile strength and elasticity of the MBm [63].

The length of the incubation period affects the quality of the composite materials. The density of fungal-based composites increased as the incubation period increased from 195 kg/m^3^ to 280 kg/m^3^ [52]. That might be due to the fact that the voids between the fibers are filled as the mycelium continues to grow and the substrate is bonded more strongly together which in turn increases the density [28]. Similarly, longer inoculation time increased mycelium composition such as chitin [71], which positively affects the compressive strength of the materials [65]. On the other hand, an extensive incubation period leads to complete degradation of the feeding substrate, which causes a decrease in compressive strength [28, 72]. The extensive growth period of sawdust above 4 weeks resulted in decreased material strength [72]. The main reason behind this might be the physical nature of the substrate [30] and its chemical contents [8].

Substrate type and strain type are also the other factors affecting the quality of the composite. The composite made from sawdust was the lowest of all substrates in water absorption capacity, and coffee husk was the highest which is strongly related to mycelium development and the density of the materials [32]. It is might due to substrate composition and substrate size [4].

The maximum density and compressive strength of MBB made from sawdust composites were 280 kg/m^3^ and 570 kPa, with 200% water absorption [32]. MBB made from mycelium and sawdust has higher compressive strength and density than bagasse [4]. The same author reported that the lower strength and density of bagasse as compared to sawdust were due to the fact that it has maximum substrate size and low mycelium penetration. Heat application during the fabrication method could increase the density and compressive strength of MBB by 2-3 folds than cold press [33, 90]. In addition, the quality of the mycelium-based composite is affected by the homogeneity of particle size and composition of raw materials [91]. The authors conclude that mycelium contains vitamin and mineral enzymes that grow well on the substrate which strongly influence the composite strength [91].

### 3.2. The Future Projection

It is mandatory to shift our economy to biomaterials to live in a sustainable environment. Further study is needed to improve the physicochemical quality of the mycelium composite. Compressive strength, density, and hydrophobicity of composite could be improved through heat application and genetic modification. Gene modification can be done through gene deletion or transformation. Furthermore, alterations in growth conditions such as light and CO_2_ levels affect hyphal density and performance, suggesting that these alterations can be used to tailor mycelium material traits. Some alkaline fungi strain can produce their own calcium carbonate and be used for self-healing of building cracks and bioconcrete. Gene transformation of such calcite-producing strain to composite-forming fungi can enhance the quality of the composite. In addition, the main concern of using composite in the construction sector is its poor water absorption. It can be improved by coating water-resistant materials.

## 4. Conclusions

The current review explores the potential of fungi-based materials in the construction sector. The composite made of fungal mycelium and the organic substrate is emission-free, nontoxic, low cost, and recyclable. Most researchers agree that fungal species belonging to the phylum Basidiomycota such as *Pleurotus ostreatus* and *Ganoderma lucidum* show better results in composite production. These species have thick mycelium, grow easily on the locally available substrate, and have high ability of cellulose degradation. Mycelium-based composite is mainly used for packaging, thermal insulation, and other different furniture. This composite shows excellent thermal stability, hydrophobic properties, and mechanical strength that can replace conventional construction materials which are nonbiodegradable, high emission, and high cost. Factors affecting the physicochemical property of the composite include type of substrate and strain, incubation time, and fabrication process.

## Figures and Tables

**Table 1:** Construction materials made of microbes.

No.	Species name	Kingdom	Products	Application	References
1	T. versicolor	Fungi	Bioblock	Thermal insulation	[28]
2	*Ganoderma lucidum*	Fungi	Block	Insulation	[12, 29]
3	*Agrocybe aegerita*	Fungi	Block	Design and architecture	[30]
4	*Aspergillus nidulans*	Fungi	Bioconcrete	Construction	[31]
5	*Trametes versicolor*	Fungi	Block	Insulation	[29]
6	*Ganoderma sessile*	Fungi	Block	Architecture	[32]
7	*Pleurotus ostreatus*	Fungi	Block	Packaging	[4]
8	*Trametes multicolor and Pleurotus ostreatus*	Fungi	Block	Construction	[33]
9	*Rhizopus oryzae*, *Phanerochaete chrysosporium*, *A. terreus*, *A. oryzae*, and *Saccharomyces cerevisiae*	Fungi	Bioconcrete	Construction	[34]
10	*T. ochracea* and *P. ostreatus*	Fungi	Board	Board	[33]
11	Not specified (white-rot basidiomycete mycelium)	Fungi	Board	Particle board	[35]
12	*Ganoderma* sp.	Fungi	Sheets	Packaging material	[36]
13	Not specified	Fungi	Sheets	Insulation panel	[36]
14	*Bacillus alkalinitrilicus* and *Bacillus licheniformis*	Bacteria	Biocement	Construction	[27]
15	*Bacillus lentus*	Bacteria	Biocement	Construction	[27]
16	*Bacillus pseudofirmus* and *Bacillus halodurans*	Bacteria	Bioconcrete	Construction	[21, 37]
17	*Bacillus sphaericus*	Bacteria	Bioconcrete	Construction	[38]
18	*Xanthomonas campestris*	Bacteria	Biopolymer	Construction	[39]
19	*Bacillus sphaericus*	Bacteria	Bioconcrete	Construction	[40]
20	*Bacillus megaterium*	Bacteria	Bioconcrete	Construction	[41]
21	*Bacillus subtilis*	Bacteria	Bioconcrete	Construction	[7, 42]
22	*Bacillus massiliensis*	Bacteria	Bioconcrete	Construction	[43]
23	*Escherichia coli*	Bacteria	Bioconcrete	Construction	[38]

**Table 2:** Mycelium-based materials with different strain and substrates.

Fungal species	Substrate type	Supplement	Moisture content (%)	Temperature (°C)	Incubation time (days)	Mold type	Drying method	Fabrication method	Target use	Compressive strength (kPa)	References
*Trametes versicolor*, *Trametes multicolor*, and *G. sessile*	Saw dust	Wheat straw	50	23	6^a^ + 6^b^	Plastic mold	Oven-dried for 48 h at 60°C	—	—	—	[32]
—	Paddy straw, fine paddy powder, and saw dust	—	—	26–27	(7–15)^a^ + 7^b^	Plastic mold	1000 C for 30–45 minutes	—	Construction materials	347	[73]
*Pleurotus ostreatus*	Sawdust, straw, and mixture	Wheat bran	67.5 ± 2.5	24 ± 1	14^a^ + 3^b^	Plastic form work	Oven-dried at 90°C for 90 min	—	Construction materials	20 to 188	[8]
*Ganoderma lucidum* and *Pleurotus ostreatus*	Cellulose	PDA	70–80	25–30	20^b^	—	60°C for 2 h	—	—	—	[74]
*P. ostreatus, Pleurotus eryngii,* and *Pycnoporus sanguineus*	Coconut powder	Wheat bran	60–70	25	(15, 30, 45)^b^	—	—	—	—	0.02 ± 0.01 to 0.04 ± 0.01	[75]
*Ganoderma lucidum*	Cotton stalk	Cotton bran	65	25	7^b^	Plastic mold	65°C for 10 hr	—	—	—	[76]
*Pleurotus ostreatus*	Sawdust	—	80	25	45^b^	Plastic mold	At 130°C for 20 and 40 min	Heat press	Composite board		[71]
*Trametes multicolor and Pleurotus ostreatus*	Sawdust and straw	—	65–70	25	14^b^	Plastic mold	—	Heat press 150°C for 20 min	—	—	[63]
*Pleurotus ostreatus*	Bagasse, sawdust, and wheat bran	—	60	25	14^a^ + 14^b^	Wooden mold	90°C for 12 hrs	10 kg load pressing	Packaging material, insulation, and furniture	6500	[4]
*Pleurotus ostreatus*, *Volvariella*, *and Polyporus squamosus*	Wood chips and hemp fiber	—	—	25	35^b^	—	Oven-dried at 70°C for 18 hrs	Compressing with spoon	Design and architecture	452	[68]
*Ganoderma* sp.	Cotton carpel	Cotton seed hull and starch	—	21	6^b^	Plastic mold	Oven-dried at 60°C for 8 hr	Hand press	Packaging	—	[36]
*P. ostreatus*, *P. citrinopileatus*, *Pleurotus eryngii*, *and G. lucidum*	Husk psyllium, flour, feathers, and textile	—	—	25	7^b^	Glass beaker	Oven-dried at 90°C for 2 hrs	Hand press	Footwear products	124.80 to 340.08	[77]
—	Saw dust and rice bran	—	—	—	33^b^	Steel mold	110–115°C for 24 hrs	—	Construction materials	4409 to 7990	[9]
*Trichoderma asperellum*, *G. lucidum*, *Agaricus bisporus*, *P. ostreatus*	Oat husk and rapeseed cake	—	—	21	14^a^ + 7^b^	Plate	40°C for 48 hrs	Oil press	Plastic	16.8 to 299.6	[44]
*G. lucidum*	Rapeseed straw	Cellulose fiber	58	30	21^b^	EPS mold	65°C for 24 hrs	Hand press	Wall insulation	845 ± 90.0	[64]
*Coriolus versicolor and Pleurotus ostreatus*	Wood chips, hemp hurd and fiber, and hemp mat	—	—	—	30^b^	Plastic mold	125°C for 2 hrs	—	Plastic	24–93	[67]
*Pleurotus ostreatus*	Soil, xanthan gum, and guar gum	Hay, glycerol, and molasses	60–70	27	20^a^ + 30^b^	Glass tank	—	—	Architectural activity	—	[78]
*Trametes versicolor*	Hardwood chips and hemp shives	—	70 ± 5	22 ± 2	—	Mold	93°C	—	Building materials	360 ± 50.0 to 520 ± 80.0	[69]
*Trametes versicolor*	Yellow birch wood veneers	—	80	28	18^b^	—	—	Hot pressing	Wood bonding	1740	[35]
—	Sawdust and millet grain	Wheat bran	—	—	14	Tubular mold	60°C for 24 h	—	Biofoam	570	[47]

^a^Incubation period before mold. ^b^Incubation period after mold.

**Table 3:** Comparison of MBB in cost, strength, density, recyclability, and manufacturing method with the conventional construction materials.

Material property	Mycelium-based materials	Polymer materials	Gypsum-based materials	Cement material
Density (kg/m^3^)	110 ± 0.01 to 330 ± 0.05^(i)^	22 to 30^(i,b)^	417–945^(c)^	1800–1950^(d)^
Cost ($/kg)	0.07–0.17^(h)^	2.1–2.3^(h)^	1.4–11^(h)^	—
Cost ($/m^3^)	19.05^(e)^	—	—	942.86^(i)^
Compressive strength (kPa)	360 ± 5 to 520 ± 8^(m)^	69–400^(l)^	60–550^(c)^	3450^(k)^
Water absorption (%)	200^(b)^	6.9^(a)^	52^(f)^	12^(k)^
Recyclability	Fully degradable^(h)^	Decades, century^(h)^	Years, decades^(h)^	None^(g)^
Raw materials	Mycelium and organic wastes or substrates^(i)^	Polymers and natural gases^(a)^	Adhesives, sawdust, and chips^(j)^	Cement and sand^(k)^
Manufacturing process	Molding and growing^(i)^	Polymerization and expansion^(h)^	Lathing, pressing, resin infusion, and milling^(h)^	Mixing, molding, and curing^(k)^

^a^[32], ^b^[85], ^c^[86], ^d^[49], ^e^[87], ^f^[7], ^g^[13], ^h^[4], ^i^[88], ^j^[89], ^k^[47], and ^l^[69].

## Data Availability

All data presented or analyzed during this study are included within this article.
